# Supplementary figures and images for: BDNF produced by cerebral microglia promotes cortical plasticity and pain hypersensitivity after peripheral nerve injury
Source: PLoS Biol. 2021 Jul 22;19(7):e3001337. doi: 10.1371/journal.pbio.3001337 (PMC8346290; doi:10.1371/journal.pbio.3001337)

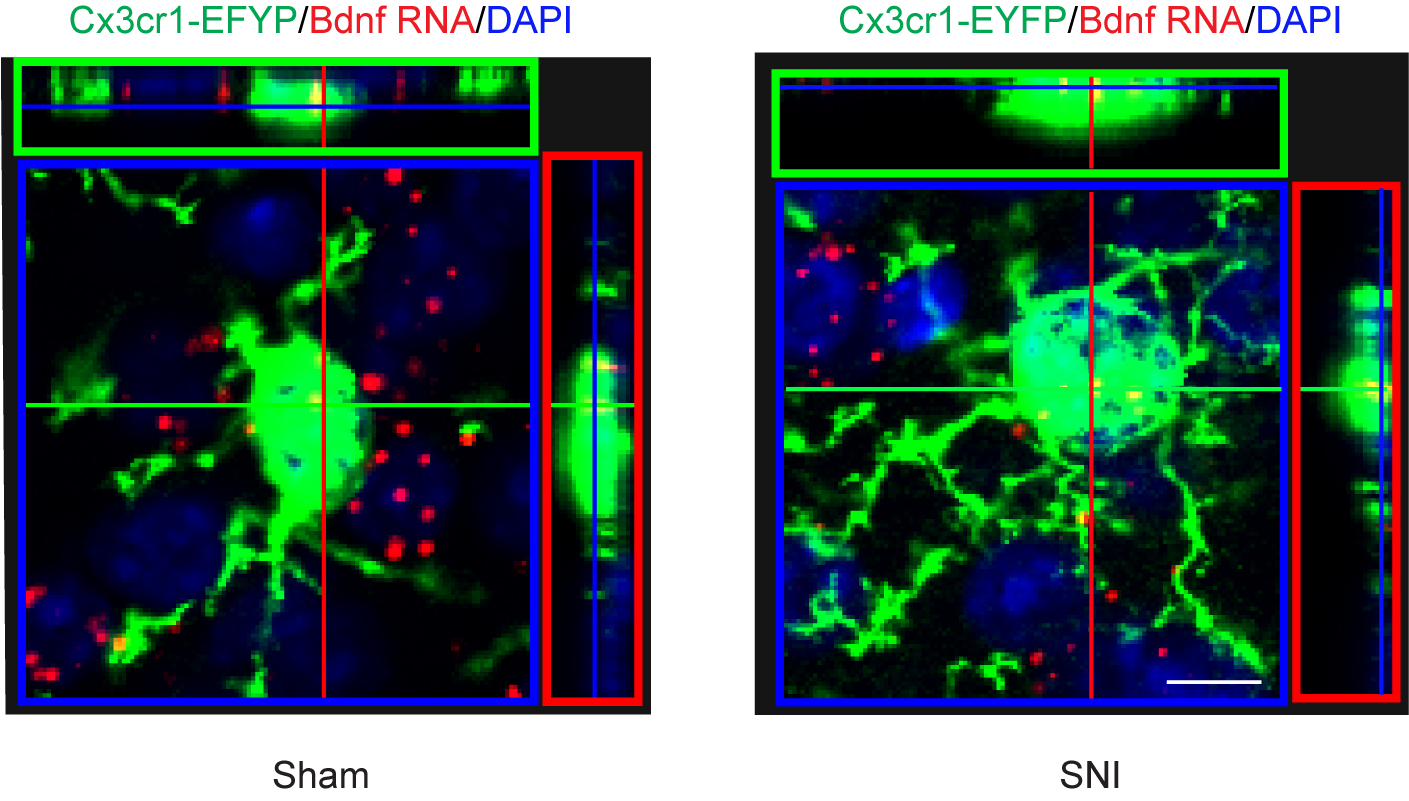

Supplement: S1 Fig — Orthogonal sections from z-stack confocal images of microglia (green), Bdnf mRNA (red), and DAPI (blue) in sham (left) and SNI (right) groups. Bdnf, brain-derived neurotrophic factor; SNI, spared sciatic nerve injury. (TIF) [file pbio.3001337.s001.tif]

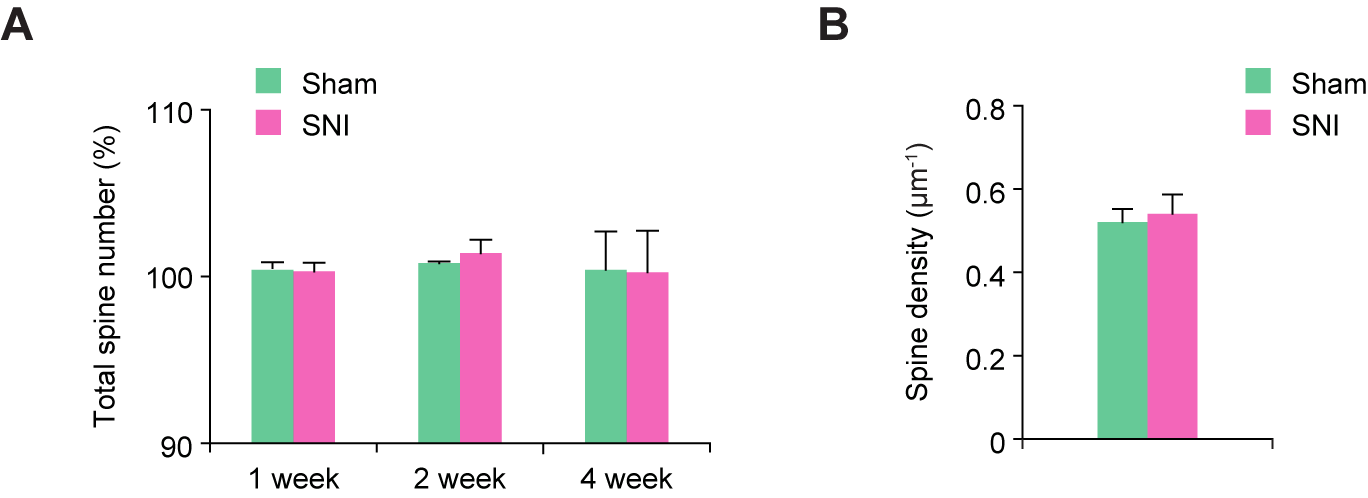

Supplement: S2 Fig — (A) The net change of total spine number in the S1 at various time points after sham or SNI surgery. (B) Density of dendritic spines on the apical tuft dendrites of L5 pyramidal neurons from SNI and sham mice (sham: 1074 spines, n = 7 mice; SNI: 1,106 spines, n = 7 mice). Data are presented as means ± SEM unpaired t test. The data underlying this figure can be found in S1 Data. L5, layer 5; SNI, spared sciatic nerve injury; S1, primary somatosensory cortex. (TIF) [file pbio.3001337.s002.tif]

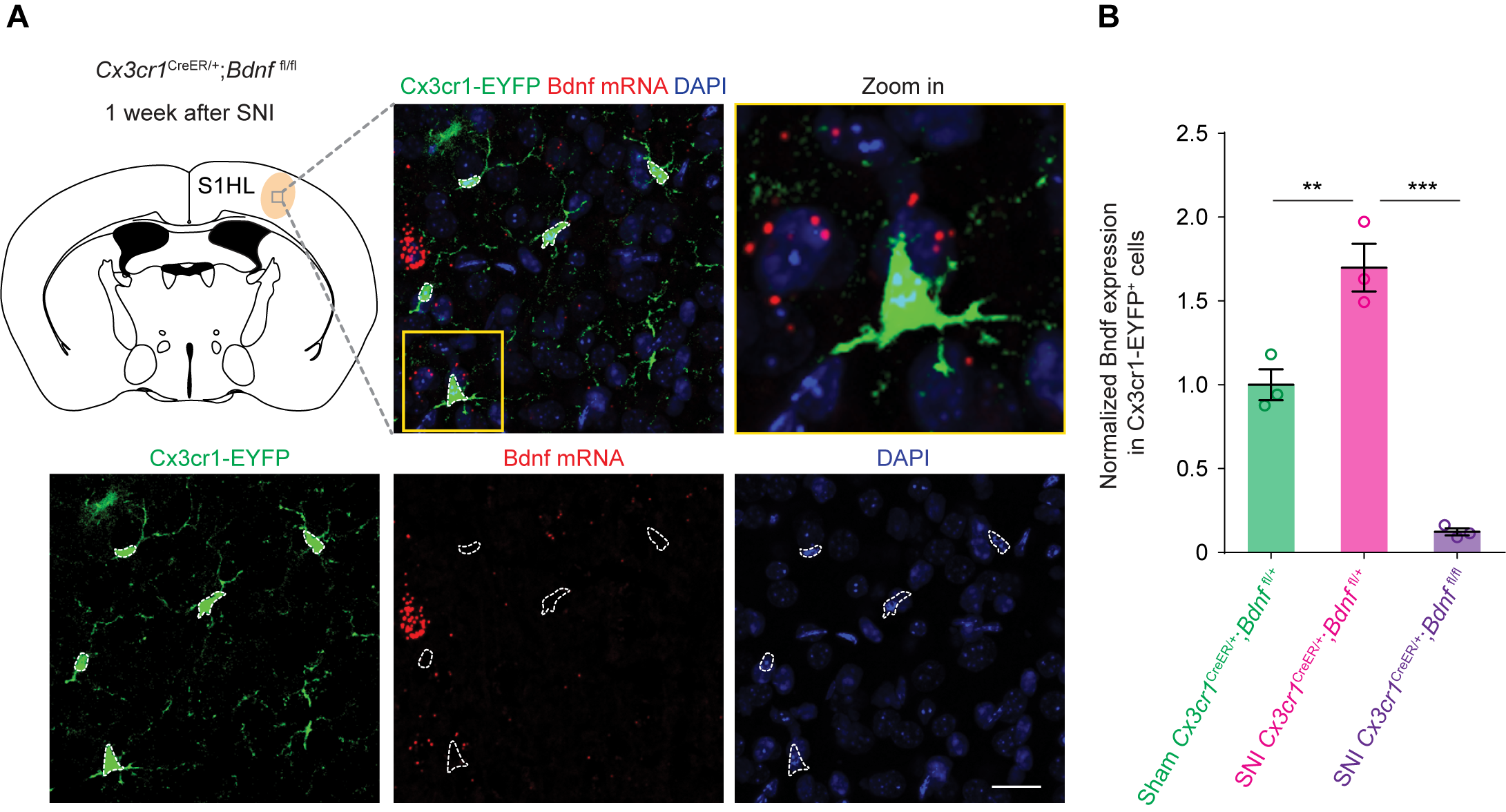

Supplement: S3 Fig — (A) RNAscope fluorescence in situ hybridization in the S1 of Cx3cr1CreER/+;Bdnffl/fl mice. Red, Bdnf mRNA probe hybridization. Green, Cx3cr1-EYFP+ microglia. Blue, DAPI. Scale bar, 20 μm. (B) Normalized levels of Bdnf mRNA in microglia (n = 3 mice per group). **P < 0.01, ***P < 0.001; by one-way ANOVA followed by Bonferroni multiple comparisons test. The data underlying this figure can be found in S1 Data. Bdnf, brain-derived neurotrophic factor; S1, primary somatosensory cortex; S1HL, hindlimb region of S1. (TIF) [file pbio.3001337.s003.tif]

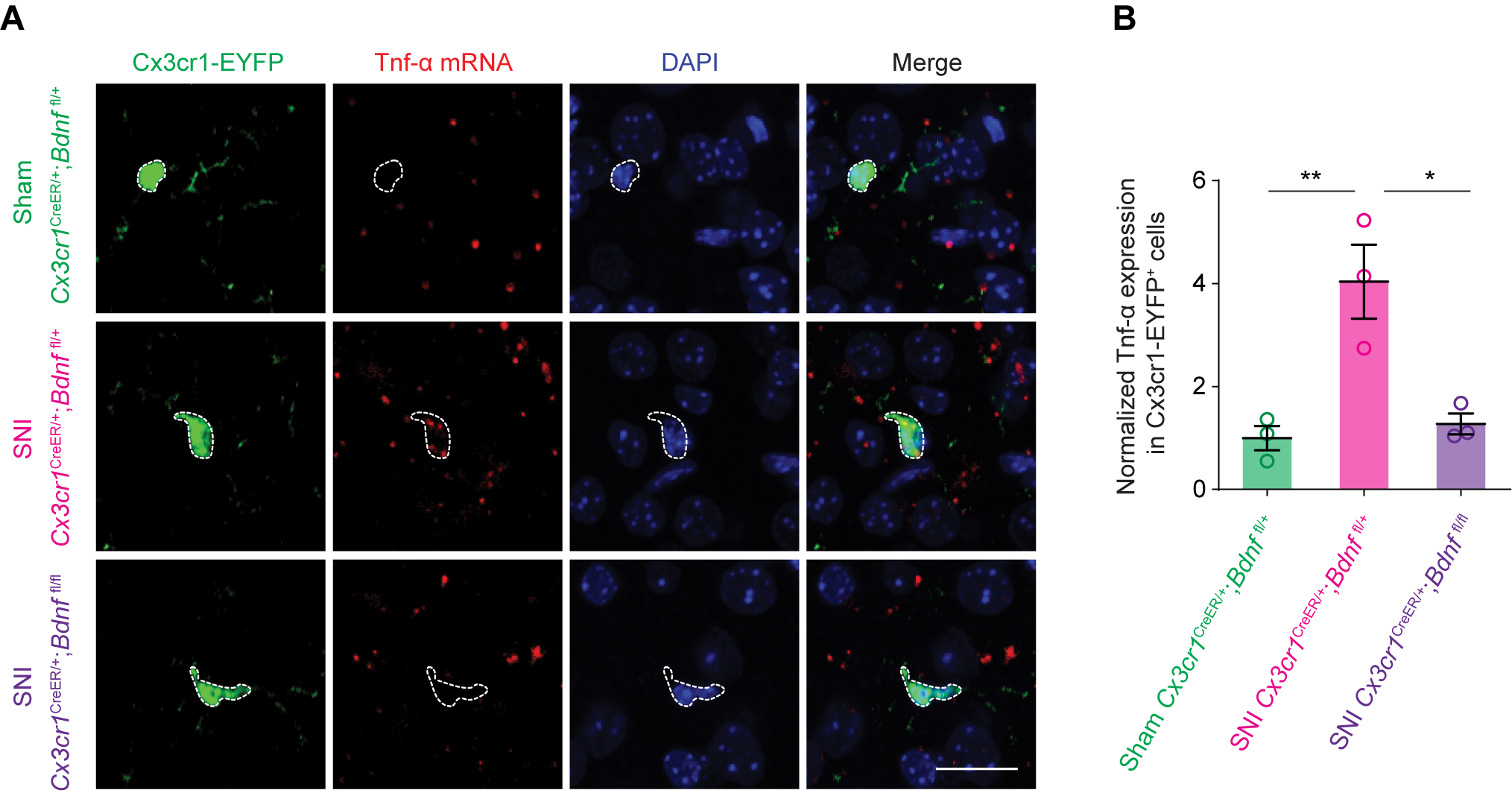

Supplement: S4 Fig — (A) RNAscope fluorescence in situ hybridization in the S1 of sham and SNI mice with or without microglial BDNF. (upper) Sham Cx3cr1CreER/+;Bdnffl/+, (middle) SNI Cx3cr1CreER/+;Bdnffl/+, (bottom) SNI Cx3cr1CreER/+;Bdnffl/fl. Red color represents Tnf-α mRNA probe hybridization. Green color indicates Cx3cr1-EYFP+ microglia. Blue, DAPI. Scale bar, 10 μm. (B) Normalized levels of Tnf-α mRNA in microglia (n = 3 mice per group). *P < 0.05. **P < 0.01; by one-way ANOVA followed by Bonferroni multiple comparisons test. The data underlying this figure can be found in S1 Data. BDNF, brain-derived neurotrophic factor; SNI, spared sciatic nerve injury; S1, primary somatosensory cortex. (TIF) [file pbio.3001337.s004.tif]

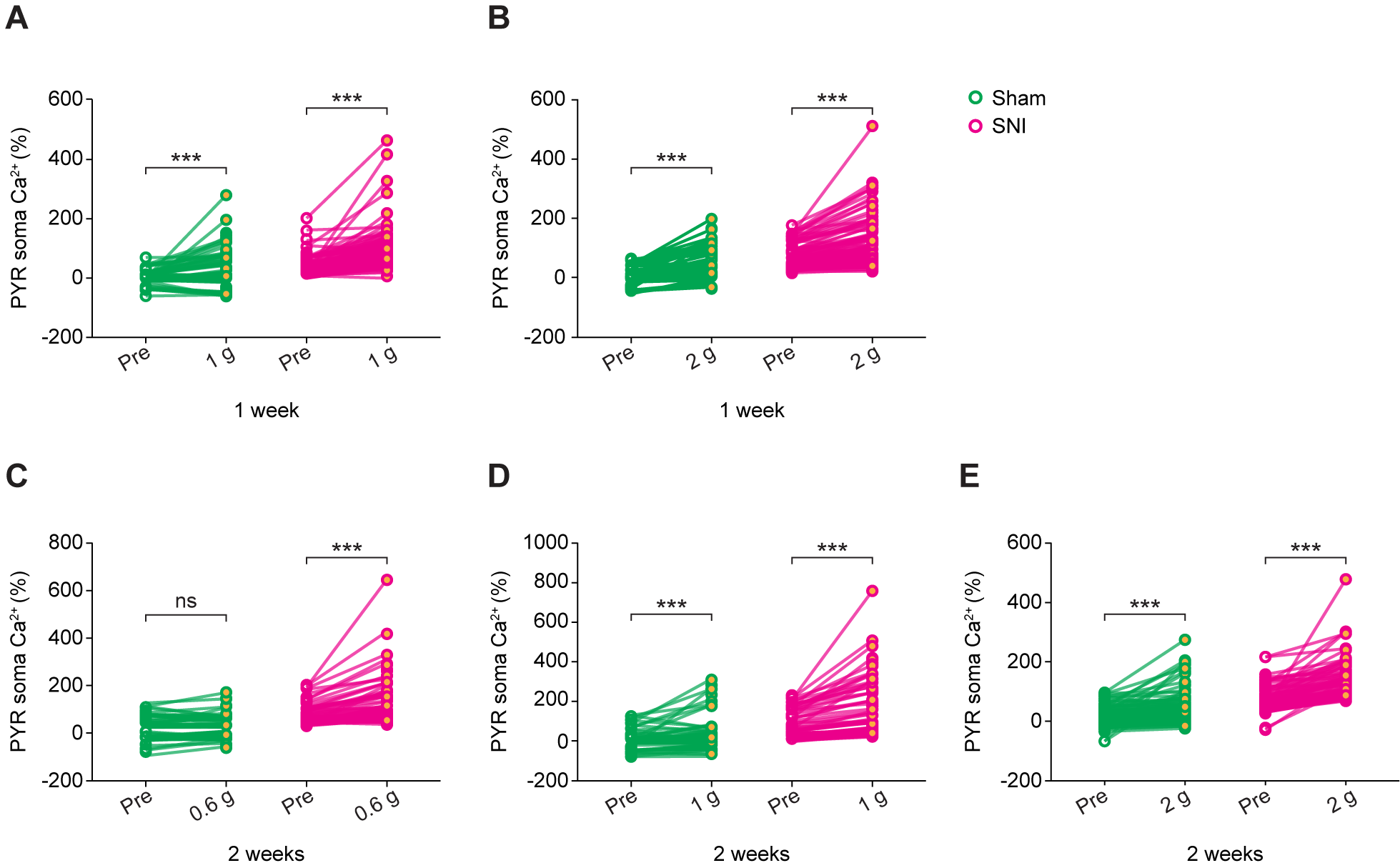

Supplement: S5 Fig — (A, B) Averaged somatic Ca2+ activity over 10 s in S1 PYR neurons before and during mechanical stimulation by 1 g (A) or 2 g (B) von Frey hair at 1 week after surgery (sham: 5 mice; SNI: 8 mice). (C–E) Averaged somatic Ca2+ activity over 10 s in S1 PYR neurons before and during 0.6 g (C), 1 g (D), or 2 g (E) paw stimulation at 2 weeks after surgery (sham: 5 mice; SNI: 5 mice). Throughout, individual circle represents data from a single cell. ***P < 0.001, ns, not significant; by paired t test. The data underlying this figure can be found in S1 Data. PYR, pyramidal; SNI, spared sciatic nerve injury; S1, primary somatosensory cortex. (TIF) [file pbio.3001337.s005.tif]
