# Supplementary material for: BDNF produced by cerebral microglia promotes cortical plasticity and pain hypersensitivity after peripheral nerve injury
Source: PLoS Biol. 2021 Jul 22;19(7):e3001337. doi: 10.1371/journal.pbio.3001337 (PMC8346290; doi:10.1371/journal.pbio.3001337)
Supplement: Attachment — Submitted filename: Response to the reviewer-R1.docx [file pbio.3001337.s007.docx]

Response to the reviewers' comments

Reviewer #1: This is a very interesting paper to investigate the role of microglial BDNF in the S1 sensory cortex for neuropathic pain after spared nerve injury (SNI). The authors used several cutting-edge techniques such as in vivo two-photon imaging of morphological changes (dendrites) and functional changes (calcium increase) and Cx3cr1CreER:Bdnfflox mice. The data showed that conditional knockout of BDNF from microglia can prevent nerve injury-induced synaptic remodeling and pyramidal neuron hyperactivity in the S1, as well as pain hypersensitivity in mice. Finally, the authors showed that S1 targeted removal of microglial BDNF via intracranial administration of tamoxifen could largely reproduce the beneficial effects of systemic BDNF depletion on cortical plasticity and mechanical allodynia. Notably, the authors examined the morphology, cell density and process motility of microglia in the S1 after nerve injury and observed no differences in the morphology and density of microglia in the S1 between SNI and sham mice (Fig 1B, C). This result is important to suggest that microglia can regulate pain even in the absence of obvious morphological changes. Overall, this is well conducted study, and the findings are significant. The following issues should be addressed before the paper is accepted for publication.

We thank the reviewer for his/her time in reviewing our study and for the positive comments. We

have now addressed all the issues raised by the reviewer with new data and revised the manuscript accordingly.

1) The RNAscope data for BDNF in figure 2 is underwhelming. There seems to be a change in BDNF mRNA between sham and nerve injury, but it seems like most of the signal is outside of microglia and it is difficult to see if the signal is above or below the microglial cells. Please describe the way the images are acquired. It will be helpful to show a confocal Z-stack to show the RNA puncta within the microglia.

As the reviewer pointed out correctly, most of Bndf mRNA puncta in Fig. 2A are outside of the microglia. This is consistent with the notion that BDNF exists in many cell types, including neurons and microglia, and Bdnf gene expression is at very low levels in microglia (Bennett et al., 2016 PNAS 113, E1738-1746; Denk et al., 2016 Cell Reports 15, 1771-1781). As suggested, we have now included confocal Z-stacks in new **Supplementary Figure 1**, showing the RNA puncta were indeed within the microglia. We have also provided detailed information on RNAscope data acquisition and analysis in the Method section.

“Fresh-frozen brains from *Cx3cr1*^CreER-EYFP^ mice were sectioned into 12-μm-thick sections. To visualize *Bdnf* or *Tnf*-α mRNA in sections of the S1, fluorescence *in situ* hybridization was performed according to manufacturers' instructions using the RNAscope Multiplex Fluorescent Reagent Kit v2 (#323100, Advanced Cell Diagnosis). Prior to probe incubation, slices were pretreated with hydrogen peroxide (10 min, room temperature), Target Retrieval Reagent (5 min, 99 °C) and RNAscope protease III (30 min, 40 °C). Slices were incubated with *Bdnf* mRNA probes (#457761, Advanced Cell Diagnostics) targeting regions between bases 662 and 1403 within the open reading frame or *Tnf*-α mRNA probes (#311081, Advanced Cell Diagnostics), and then hybridized to Opal 690 (PerkinElmer FP1497001KT) for visualization. Positive control probes (#312481, Advanced Cell Diagnostics) and negative control probes (#310043, Advanced Cell Diagnostics) were used in the parallel experiments to confirm specificity of hybridization. To visualize microglia together with mRNA puncta, brain slices were incubated at room temperature overnight with a primary antibody against GFP (1:250, Abcam) and then for 2 h with Alexa Fluor 488-conjugated goat anti-rabbit IgG (1:500, Life Technologies). After rinsed with PBS, the sections were incubated with DAPI solution (1:50,000) and mounted with ProLong Gold Antifade Mountant (Invitrogen).

The images were acquired with a 40× objective at a zoom of 3.0 using a Zeiss LSM 800 confocal microscope. For each microscopic field of view (66 μm × 66 μm), a stack of 18 image planes at 0.5 μm increments along the z-axis were collected using the ZEN software, yielding a full three-dimensional data set. Microglia were identified from three-dimensional image stacks [32]. A total of 180–250 microglia from 4 animals per condition were examined for the presence of *Bdnf* mRNA and the number of puncta per cell was quantified using the NIH ImageJ software. The negative controls were used to define the background during data analysis.”

2) It is difficult to see Bdnf mRNA expression by 60% of microglia. In Fig. 2A with SNI, it looks only one microglial cell showing clear puncta in a field of 5 microglia.

We have now replaced the immunofluorescence pictures in Fig. 2A with new pictures, in which 4 out of 5 microglial cells showing Bdnf mRNA puncta. We also show one such positive cell in a higher resolution. The new images are more representative of the quantification result in Fig. 2D, showing Bdnf mRNA expression by 60% of microglia. We thank the reviewer for pointing this out.

3) If you only have 2 puncta per microglial cell, how is the background defined for RNAscope? I wonder if you did RNAscope using Cx3cr1^CreER/+^:Bdnf^fl/fl^ mice.

For RNAscope experiments, we have included both positive and negative control probes. The negative controls were used to define the background during data analysis. We have now provided the details of RNAscope analysis in the **Method** section.

We have now performed RNAscope experiments using Cx3cr1^CreER/+^:Bdnf^fl/fl^ mice. Consistent with previous reports that BDNF can be specifically removed from microglia in Cx3cr1^CreER/+^;Bdnf^fl/fl^ mice (Parkhurst et al, 2013 Cell 155, 1596–1609), we did not detect Bdnf mRNA puncta in microglia of Cx3cr1^CreER/+^:Bdnf^fl/fl^ mice after tamoxifen administration. These new data are presented in new **Supplementary Fig. 3**.

4) Fig. 2E. It is unclear how many animals were used for the quantification. How many sections were evaluated per animal? I wonder if the sample size is based on the number of sections or number of microglia instead of number of animals?

We have now provided detailed information for RNAscope analysis in both figure legends and the Methods section. For RNAscope analysis, on average, 9 sections were evaluated per animal. In Fig. 2E, each data point represents one microglia. The sample size is based on the number of microglia. We analyzed a total of 180–250 microglia from 4 animals per condition.

5) Male mice were used in this study. Spinal microglial BDNF signaling was shown to be male dominant in neuropathic pain. I wonder if there is sex difference in cortical microglia.

We thank the reviewer for raising this interesting question. We have now performed new experiments on the sex difference in cortical microglia. Specifically, we isolated microglia from the contralateral S1 using fluorescence-activated cell sorting and measured the mRNA levels of various gliotransmitters before and after SNI in both male and female mice. Our quantitative PCR results indicate that SNI in male mice markedly increased *Bdnf* mRNA expression by 3- and 9-fold over 1 and 2 weeks, respectively (**new** **Fig. 1F**). The mRNA levels of *Tnf*-α, *Il*-6, and *Il*-1β in microglia were also significantly elevated in males 2 weeks after SNI. By contrast, female mice exhibited only a transient elevation of *Bdnf*, *Tnf*-α and *Il*-6 transcript in microglia (**new** **Fig. 1F**). Two weeks after surgery, there was no difference in the mRNA expression of various gliotransmitters between SNI and sham groups in females. These results indicate that cortical microglia respond to peripheral nerve injury in a sex-dependent manner, consistent with the notion that the involvement of microglial signaling in neuropathic pain is sexually dimorphic.

Other comments:

Discussion: "Following nerve injury or inflammation in peripheral tissues, BDNF is upregulated in the ipsilateral dorsal root ganglia, in the dorsal horn of the spinal cord, as well as in supraspinal regions and various cortical areas such as the S1 and the ACC (ref 37-39)". The authors may check earlier studies showing BDNF upregulation in DRG neurons in inflammatory pain and neuropathic pain (PMID: 10430952; PMID: 11425916).

We have now included these two studies in the reference. We thank the reviewer for the suggestion.

Discussion: "targeting microglia-neuron signaling pathways in the somatosensory circuits could be an effective strategy to prevent and treat chronic neuropathic pain". It will be difficult to target this signaling pathway in the S1 cortex without causing side effects. This brain region is also difficult to access using non-invasive method. BDNF also has beneficial effects. Additional discussion will help the paper.

We thank the reviewer for raising these good points. We have now removed the sentence "targeting microglia-neuron signaling pathways in the somatosensory circuits could be an effective strategy to prevent and treat chronic neuropathic pain" from the Discussion. We have now included additional discussion, with a focus on microglial sex dimorphism and microglia-neuron signaling.

Reviewer #2: The present investigation by Huang et al was undertaken to identify a role of microglia in abnormal cortical plasticity associated with the spared nerve injury (SNI)-induced neuropathic pain. In this study, authors noted no change in the cortical microglia morphology and density, and a mild change in the motility of microglia processes post-SNI. Also, it was reported that the expression of Bdnf mRNA was upregulated in microglia of the contralateral primary somatosensory cortex (S1), assessed using qPCR and RNAscope fluorescence in situ hybridization. In a series of experiments involving the use of transgenic mice and two-photon imaging authors observed that the SNI mice show an increase in synapse remodeling (increase in elimination and formation of dendritic spines) and somatic Ca2+ responses in L5 neurons. However, upon genetic depletion of BDNF, synaptic remodeling, neuronal hyperactivation and mechanical allodynia was prevented. Based on the findings presented here, authors propose that microglia-neuron signaling involving BDNF is important for abnormal cortical plasticity pertaining to chronic pain caused by traumatic peripheral nerve injury.

Major Comments to the Authors:
1.    Microglial BDNF involvement in SNI pain behavior has been reported to be restricted to male specific pain pathways (Sorge 2015, Zhou 2019, Saika 2020). Therefore the authors must test the necessity of microglial BDNF in S1 cortex in female mice with SNI. If there is no sex difference, this would be a highly intriguing finding. The Authors should also consider local tamoxifen-induced knockout of BDNF in the spinal cord to ascertain if spinal microglial-derived BDNF is dispensable for pain behaviors in male and female mice.

We thank the reviewer for raising the important question regarding microglial functions in male and female mice. As suggested by the reviewer, we have now examined the expression of cortical microglia BDNF in male and female mice with SNI. Specifically, we isolated microglia from the contralateral S1 cortex using fluorescence-activated cell sorting and measured the mRNA levels of Bdnf before SNI, 1 week and 2 weeks after SNI in both male and female mice. Our qPCR results reveal a progressive increase in cortical microglial *Bdnf* in male mice: *Bdnf* mRNA was increased by 3-fold at 1 week after SNI and 9-fold at 2 weeks after SNI relative to the pre-SNI base line (new **Fig. 1F**). By contrast, female mice only exhibited a transient elevation of Bdnf transcript in cortical microglia at 1 week after SNI. Two weeks after surgery, there was no difference in Bdnf mRNA expression between SNI and sham groups in females (new **Fig. 1F**), though SNI induced pain behaviors last for at least 1 month in mice. These results indicate cortical microglia respond to peripheral nerve injury in a sex-dependent manner, and the involvement of cortical microglial Bdnf signaling in chronic neuropathic pain is likely male-biased. These results are also consistent with previous studies by Sorge et al (Nature neuroscience. 2015;18(8):1081-3) showing that systemic deletion of microglia BDNF, including cortical microglia, has effects on pain hypersensitivity in male, but not female, mice.

In the revised manuscript, we have presented the data related to sex difference in cortical microglia Bdnf and Tnf-alpha expression in the new **Fig. 1F**. We have also provided additional discussion on the sex difference of spinal microglial signaling as it relates to chronic pain. Because the goal of the present study is to determine whether in male mice, cortical microglia may increase BDNF expression after peripheral nerve injury and also contribute to the somatosensory cortical plasticity associated with neuropathic pain, we did not perform local tamoxifen-induced knockout of BDNF in the spinal cord. We hope the reviewer would agree with us that the experiments of testing spinal microglial BDNF in male and female mice with SNI are informative but out of the scope for the current studies.

2.    Molecular assessment was not performed on tamoxifen treated CX3CR1-BDNF(fl/fl) mice to determine whether Bdnf mRNA was indeed depleted. As in Figure 1F, the authors need to perform qPCR for Bdnf expression in isolated microglia from the knockout mice. Additionally, the Authors need to measure IL-6, IL-1beta and TNF-alpha as well in the knockout cells. Liu 2017 has shown that brain TNF-alpha from microglia regulates synaptic plasticity and spine formation after SNI, and Rossetti 2019 has shown that IL-1beta in some parts of the brain increases in expression in females after BDNF knockout. Therefore it is necessary for the Authors to assess if microglia from tamoxifen treated CX3CR1-BDNF(fl/fl) mice express these signaling transcripts differentially as any of these molecules may provide a mechanistic explanation for the observed dendritic spine remodeling.

As suggested, we have now performed molecular assessments on Bdnf and Tnf-alpha expression in tamoxifen-treated Cx3cr1^CreER/+^;Bdnf^fl/fl^ mice. Using RNAscope fluorescence *in situ* hyberization, we confirmed that Bdnf mRNA was indeed depleted from microglia in the knockout mice (new **Supplementary Fig. 3**). We have also measured the mRNA level of TNF-α in the knockout mice (new **Supplementary Fig. 4**). Interestingly, we found that SNI increased the expression of TNF-α in cortical microglia in Cx3cr1^CreER/+^:Bdnf^fl/+^ mice (with microglia BDNF), but not in Cx3cr1^CreER/+^;Bdnf^fl/fl^ mice (without microglia BDNF). Given the important role of TNF-α in synaptic plasticity (Stellwagen et al, Nature. 2006;440(7087):1054-9; Garre et al, Nat Med. 2017;23(6):714-22), these results suggest that microglia BDNF may affect neuronal plasticity associated with neuropathic pain through the regulation of glial TNF-α. We have now mentioned such a possibility in the discussion.

Unfortunately, we could not perform the IL-1beta experiments suggested by the reviewer. Over the past 1 year, there has been a substantial reduction of mouse colony in our laboratory due to the Covid 19 pandemic. As a result, we have had difficulties to obtain enough number of mice of desired genotypes (Cx3cr1^CreER/+^;Bdnf^fl/fl^ and Cx3cr1^CreER/+^;Bdnf^fl/+^) to examine the effects of BDNF depletion on SNI-induced IL-1beta elevation. We thank the reviewer for suggesting these important experiments.

3.    The RNAscope assessment of Bdnf mRNA puncta is not convincing. The Bdnf mRNA puncta are distributed across the images but very few are localized over microglia somata. The'yellow' puncta could represent neuronal, or other, sources overlapping the soma of the microglia. The Authors need to show the side profiles for these images to show the readers whether the signals are present inside the soma of microglia. Additionally, the Authors need to perform RNAscope in brain sections from the microglia Bdnf knockout. It is surprising this was not done as it would provide irrefutable evidence supporting the validity of using RNAscope to detect Bdnf mRNA in microglia.

As the reviewer pointed out correctly, most of Bdnf mRNA puncta in Fig. 2A are outside of the microglia. This is consistent with the notion that BDNF exists in many cell types, including neurons, astrocyte and microglia. Under physiological conditions, neurons are the major source of BDNF, while Bdnf mRNA expression is at very low levels in microglia (Bennett et al., 2016 PNAS 113, E1738-1746; Denk et al., 2016 Cell Reports 15, 1771-1781). Consistently, our RNAscope data show that Bdnf transcript can only be visualized in a fraction of microglia in the S1 and most of these cells possess only a few Bdnf mRNA puncta under physiological conditions (**Fig. 2A**). Despite this low basal level of microglial Bdnf mRNA, we did observe that the levels of microglia Bdnf mRNA in S1 cortex was significantly elevated after SNI, as shown by both qPCR (new **Fig. 1F**) and RNAscope data (**Fig. 2D, E**).

As suggested, we have now included confocal Z-stacks in new **Supplementary Figure 1**, showing the side profiles of EYFP-labelled microglia indicating that the mRNA signals are indeed inside the soma of microglia.

We have also performed RNAscope in brain sections from the microglia Bdnf knockout mice. No *Bdnf* mRNA was detected in the somas of microglia in tamoxifen-treated *Cx3cr1*^CreER/+^;*Bdnf*^fl/fl^ mice, consistent with previous reports that BDNF can be specifically removed from microglia using *Cx3cr1*^CreER/+^;*Bdnf*^fl/fl^ mice (Parkhurst et al, 2013 Cell 155, 1596–1609). These new data were presented in new **Supplementary Fig. 3**.

4.    Regarding somatic Ca^2+^ recordings. The figure panels for the analyses unclear. For example, in figure 5C, it is not clear how percent change is calculated given that the 'baseline' is changing. Which part of these traces are used as baseline? Is the measured change the distance of peaks vs baseline? Why not calculate area under the curve? In the figure 5D, does 0% mean no change from baseline (ie, 100% equal to baseline)?  For evoked Ca^2+^ measurements, it is mentioned in the method that the 10s prior to evoked stimulation is used as baseline. However, in all the figures showing raw traces during evoked stimulation, the baselines are cut off. The Authors should show at minimum a full 20s of traces prior to the evoked stimulation so that the readers can see what the baseline looked like. Area under the curve would seem to be a better measure of Ca^2+^ signal, doesn't require establishing a baseline and is easier to interpret.

As suggested, we have now presented a full 20-s baseline Ca^2+^ traces prior to the mechanical stimulation in Fig. 5E.  We have also provided additional information in the Method about somatic Ca^2+^ analysis. The fluorescence time course (F) of each neuron was measured by averaging all pixels within the ROI, after the correction of background. Ca^2+^ response (Δ*F*/*F*_0_) was calculated by (*F*-*F*_0_)/*F*_0_, where *F_0_* is the baseline fluorescence signal averaged over a 20-s period corresponding to the lowest fluorescence signal during the 2.5-min recording period. *F_0_* was the same for resting and stimulus conditions as the same neuron was analyzed. The mean AUC (area under the curve) of calcium response curve (*ΔF/F_0_*) over 2.5-min was presented as the somatic Ca^2+^ activity under various conditions in Fig. 5D-I.

5.    Regarding Figure 8D, E. The Authors need to show spontaneous Ca2+ traces akin to Figure

As suggested, we have now shown spontaneous Ca^2+^ traces of previous Fig. 8D, E (current Fig. 8E, F) in new **Fig. 8D**.

6.  Did local S1 depletion of Bdnf affect spontaneous Ca2+ transients in S1 neurons?

Local S1 depletion of Bdnf did affect spontaneous Ca^2+^ transients in S1 neurons after SNI. We have now provided these data in new **Fig. 8D-F**. We thank the reviewer for raising this question.

Other comments to the Authors:

1.    Typo (text says 'BNDF' - it should be 'BDNF') under intro section - paragraph 3, results fig 7 description

Corrected.

2.    Regarding Figure 1F. It is well known that activity of microglia affects expression of actin and actin regulatory molecules. The use of actin mRNA as a single reference for microglia gene expression is questionable. It is commonly accepted to use Hprt1, 18S, Eef2 or Abt1 as reference genes for microglia/macrophage cells. The authors should consider repeating the experiments in figure 1F with a more appropriate reference gene(s).  Additionally, the figure includes the double normalized Sham groups (ddCT method). This is unnecessary and only the SNI can be displayed with a dashed line across the value of 1 to depict the doubly normalized sham values. Ideally the authors should display only the dCT values (singly normalized) for both the sham and SNI groups in order to show the readers the degree of variation present in the Sham groups and also the degree of detectability for each of the transcripts.

As suggested, we have now repeated qPCR experiments using Eef2 as the reference gene for sorted microglia cells and presented the singly normalized values for both the sham and SNI groups. We thank the reviewer for pointing this out.

3.    Regarding Figure 7B. Why is there no background signal in spinal cord DsRed panel? It seems the exposure was set far too low.  Also, Figure 7c, the spinal cord section seems to be a thoracic segment, or at least does not appear to be L4/5 level which is expected to be shown for a hindpaw SNI model.

We have now presented a high-resolution picture in Fig. 7B, in which the background signal in spinal cord DsRed panel is evident. Because this set of experiment did not involve SNI surgery, we did not specifically choose L4/5 level of the spinal cord for image collection.

4.    Regarding Figure 8. If i.t. injection of tamoxifen can deplete spinal microglial Bdnf, then the Authors should test pain behavior to ascertain if local spinal microglial Bdnf is required for pain behavior. This would add considerably to establishing necessity versus sufficiency of S1 microglial Bdnf involvement in SNI pain signaling.

Previous studies have shown that spinal microglial BDNF is important for pain hypersensitivity (Coull et al, Nature. 2005;438:1017-21; Sorge et al, Nature neuroscience. 2015;18(8):1081-3; Zhou et al, Cell Rep. 2019;27(13):3844-59 e6). Therefore, we expect that local spinal microglial Bdnf would be required for pain behaviors after SNI. The goal of our study is to determine whether cortical microglia may increase Bdnf expression after peripheral nerve injury and also contribute to the somatosensory cortical plasticity associated with neuropathic pain. Our data indicate that S1 microglial Bdnf involvement is necessary for SNI pain development. We did not perform local tamoxifen-induced knockout of BDNF in the spinal cord to test whether S1 microglial Bdnf involvement is sufficient for SNI-induced pain. Given the importance of spinal microglial BDNF, it is likely that both spinal and S1 microglial Bdnf involvement are important for pain development after SNI. We hope the reviewer would agree with us that the experiments of testing whether spinal microglial BDNF is required for pain hypersensitivity after SNI is informative but not essential for the current studies.

Reviewer #3: This study by Huang et al. reveals that microglia play an important role in the structural and functional changes taking place in the primary somatosensory cortex (S1) after peripheral nerve injury in mouse. Following peripheral nerve injury, microglia in the S1 maintain a surveillant morphology and normal density but they up-regulate their mRNA expression level of brain-derived neurotrophic factor (BDNF). Using transcranial in vivo two-photon imaging and Cx3cr1CreER:Bdnfflox mice, the authors show that conditional knockout of BDNF from microglia prevents nerve injury-induced synaptic remodelling and pyramidal neuronal hyperactivity in the S1, as well as pain hypersensitivity in mice. In addition, S1 targeted removal

of microglial BDNF largely recapitulated the beneficial effects of systemic BDNF depletion on cortical plasticity and allodynia. Together, these findings reveal an unexpected role of cerebral microglial BDNF in somatosensory cortical plasticity and pain hypersensitivity.
The study uses cutting-edge mouse models as well as technical approaches. It is elegantly designed and conducted.

I only have a few important requests for revision to strengthen the findings:
1) The rationale for performing experiments in male mice only should be explained, especially considering the important sexual dimorphism in microglial BDNF function that was reported (e.g. in models of neuropathic pain).

As suggested by the reviewer, we have now provided the rationale for performing experiments primarily in male mice in the revised Introduction.

“Increasing evidence suggests that immune cells play active roles in neuronal plasticity and chronic pain, and their involvement in pain modulation appears to be sexually dimorphic [17-19]. For example, microglial signaling has been linked to neuropathic pain hypersensitivity in male rodents, while T cells are thought to be involved in females [20]. As resident immune cells, microglia occupy all regions of the mammalian central nervous system (CNS), including brain and spinal cord. Following peripheral nerve injury, microglia in the dorsal horn, the sensory processing region of the spinal cord, transform into reactive phenotypes, producing and releasing a variety of substances that modulate spinal neuron function. Among those, brain derived neurotrophic factor (BDNF) has been shown to drive disinhibition and hyperexcitation of dorsal horn neurons, ultimately resulting in pain hypersensitivity [21]. Consistently, pain hypersensitivity in male mice is reduced by depletion of spinal microglia or microglial BDNF [22-25]. Despite the crucial role of microglia signaling in spinal mechanisms of chronic pain, there is less understanding about the function of brain microglia in somatosensory plasticity.”

Additionally, we have now included new experiments on the sex difference in cortical microglia. Specifically, we isolated microglia from the contralateral S1 using fluorescence-activated cell sorting and measured the mRNA levels of Bdnf and various cytokines before and after SNI in both male and female mice. Quantitative PCR results indicate that SNI in male mice markedly increased *Bdnf* mRNA expression by 3- and 9-fold over 1 and 2 weeks, respectively (new **Fig. 1F**). The mRNA levels of *Tnf*-α, *Il*-6, and *Il*-1β in microglia were also significantly elevated in males 2 weeks after SNI. By contrast, female mice exhibited a transient elevation of *Bdnf*, *Tnf*-α and *Il*-6 transcript in microglia (new **Fig. 1F**). Two weeks after surgery, there was no difference in the mRNA expression of various gliotransmitters between SNI and sham groups in females. These results indicate that cortical microglia respond to peripheral nerve injury in a sex-dependent manner, consistent with the notion that the involvement of microglial signaling in neuropathic pain is sexually dimorphic.

2) Throughout the manuscript, the microglial nomenclature should be revised based on the seminal findings, notably from co-author Wenbiao Gan, that microglia are not quiescent at steady state. Ramified or quiescent should thus be replaced by surveillant.

We have now used “surveillant” replace “ramified” and “quiescent” in the text.

3) The LSD posthoc test is not stringent enough and it would be strongly recommended to use Bonferroni or Tukey instead.

We have now re-performed post hoc analysis with Bonferroni or Tukey test. We thank the reviewer for the suggestion.

4) The discussion is the weakest part of this manuscript. It would be important to elaborate further on the limitations of the study, including models used (e.g. Thy1-YFP mice display different responses to spinal injury versus wild-type mice; tamoxifen modulates the immune system including microglia)

As suggested, we have now expanded the Discussion section of the manuscript. Specifically, we have included one paragraph discussing the sex difference in microglial signaling in pain hypersensitivity and elaborated further on the limitations of the work.

“Recent findings have revealed that the contributing role of microglia in chronic pain is male-biased [36]. In the spinal cord, activating toll-like receptor 4, which is primarily expressed by microglia, by the agonist lipopolysaccharide, causes pain behavior in male but not female mice [37]. In rodent models of chronic inflammatory and neuropathic pain, there is a male-specific activation of microglial signaling in the spinal cord which includes the upregulation of P2X4 receptors [38], phosphorylation of p38-mitogen-activated protein kinase [39], and subsequent synthesis and release of BDNF [23]. Whether such male-biased microglial pathways also exist in the brain, contributing to pain hypersensitivity, is less investigated. In the present study, we showed at the mRNA level that microglia in the S1 displayed a lasting increase of gliotransmitters after peripheral nerve injury. Two weeks after injury, the increase of microglia *Bdnf*, *Tnf*-α, *Il*-6, and *Il*-1β occurred only in males, indicating a sex difference in brain microglial responses to peripheral nerve injury, which is consistent with recent transcriptomic findings that microglia in the healthy adult brain is sexually differentiated [40,41].”

We agree with the reviewer that tamoxifen modulates the immune system including microglia. Because we administrated tamoxifen twice in both sham and SNI *Cx3cr1^CreER/+^*;*Bdnf*^fl/fl^ and *Cx3cr1^CreER/+^*;*Bdnf*^fl/+^ male mice, and waited for 1 month before we performed SNI/sham surgery and data collection, the effect of tamoxifen on immune system is likely to be transient and unlikely to alter long-term effect of microglial BDNF in pain development. We acknowledged that the precise underlying signaling pathways underlying microglial BDNF functions are complicated and remain to be investigated in SNI and other pain models.

5) It would be interesting to provide further discussion on how, by which mechanisms downstream of BDNF, microglia modulate the response to peripheral nerve injury.

We have now expanded the discussion on the mechanisms of BDNF signaling in SNI-induced neuronal plasticity.

“The mechanisms underlying microglial BDNF-dependent neuronal plasticity remain to be determined. In the spinal cord, nerve injury increases ATP release from dorsal horn neurons, which activates P2X4 receptors of spinal microglia [51-53], resulting in the phosphorylation and activation of p38-MAPK and subsequently the expression and release of BDNF. Secreted BDNF binds neuronal TrkB receptors, leading to the downregulation of potassium chloride cotransporter, which decreases the efficacy of GABA_A_-mediated inhibition, resulting in the increased excitability of spinal neurons [21]. In the cortex, microglial processes are highly motile [54], located in close proximity to synaptic terminals, and have been implicated in synapse formation and pruning [55,56]. Under conditions of neuropathic pain, there is a substantial elevation of synaptic activity in apical dendrites of S1 pyramidal neurons [13]. Because ATP, a robust chemoattractant of microglial processes, can be released from nerve terminals [57], enhanced sensory input into the S1 may recruit local microglia processes via an ATP-dependent mechanism to release BDNF. Microglial BDNF could facilitate structural and functional remodeling of cortical neurons via similar mechanisms as demonstrated in the spinal cord. In addition, we found that microglia in the S1 upregulate the expression of proinflammatory cytokines, including TNF-α, after peripheral nerve injury, and depletion of microglial BDNF prevents the increase of TNF-α. Given the important role of TNF-α in synaptic plasticity [58, 59], it is also possible that cortical microglial BDNF promotes the neuronal plasticity in the S1 through the regulation of glial TNF-α.”
